# Application of Fuzzy Clustering Model in the Classification of Sports Training Movements

**DOI:** 10.1155/2022/4308283

**Published:** 2022-05-31

**Authors:** Bo Song

**Affiliations:** School of Physical Education, Liaocheng University, Liaocheng, Shandong 252000, China

## Abstract

In order to accurately analyze the movements of sports training using artificial intelligence techniques, an improved fuzzy clustering model is proposed in this study. The fuzzy C-means is used to granulate the multilabel space, and the correlation degree between different variable labels is obtained through information gain. Aiming at the problem of multilabel information classification, an appropriate membership function is selected, which is used to map all information samples and obtain the membership degree of its category. Considering the slow training efficiency of fuzzy support vector machine, the clustering method is used to optimize the fuzzy support vector machine, establish the optimal hyperplane, and complete the classification according to their respective attributes in high-dimensional space. Finally, the proposed algorithm and other algorithms are experimentally compared on the published KTH and Weizmann human behavior data sets. Experimental results show that the proposed method is effective and robust.

## 1. Introduction

Human motion recognition is one of the research hotspots in the field of computer vision [1]. With the progress of related technology, the human motion recognition algorithm based on deep learning has achieved a good recognition effect when the sample size is sufficient [2]. However, in the case of small or even missing sample size, there is little research on how to identify new actions.

For the recognition of training action, in addition to the ability of the selected features to describe the action, we should also consider the feasibility of subsequent action evaluation. Training movement evaluation is to evaluate the standard degree of movement execution by capturing the relative movement between each link of the human body [3, 4]. The overall displacement of human body is an irrelevant interference feature, such as the traveling displacement in the horizontal direction during running and the vertical height during jumping. Therefore, extracting the relative motion characteristics between human rings in training movements is not only conducive to distinguish similar movements but also create conditions for action evaluation. However, in the previous action recognition methods based on traditional feature extraction, the influence of irrelevant displacement on action recognition in the process of human motion is rarely considered [5, 6].

Accurate analysis and prediction of human movement can provide effective data support for sports training. By obtaining the relevant data of human movement, combined with the standard database data to correct the details of athletes' movements, we can improve the athletes' sports level. With the continuous progress of computer science and technology, the artificial intelligence technology is gradually matured. A deep neural network (DNN) can automatically learn data features to find sparse and distributed big data features [7]. However, the latest convolution technology cannot be directly applied to capture human motion data. In order to capture human motion data, the convolution filter needs to cover the whole range of human joints so that convolution occurs only in the time direction [8].

With the rapid development of computer vision and image processing technology, the intelligent training system based on visual information processing is gradually applied to sports training [9] and rehabilitation medical field [10, 11]. At present, most of the existing sports training guidance schemes relying on intelligent devices lack effective monitoring of the sports process and cannot give feedback and suggestions, which is not conducive for beginners to master movements [12]. To solve the above problems, this study designs a fuzzy clustering model, which improves the characteristics of classification labels through the fuzzy C partition method and information gain and realizes the purpose of information classification optimization by relying on a fuzzy support vector machine and clustering.

The innovations and contributions of this study are listed as follows:

Fuzzy C-means was used to granulate multilabel space, and the correlation degree between labels with different variables was obtained by information gain.
Aiming at the problem of multilabel information classification, an appropriate membership function is selected, which is used to map all information samples and obtain the membership of its category.
(3)Considering the slow training efficiency of fuzzy support vector machine, the clustering method is used to optimize the fuzzy support vector machine, establish the optimal hyperplane, and complete the classification according to their respective attributes in high-dimensional space.

The final results are verified on the public data sets of KTH [13] and Weizmann [14], and the effectiveness of this method in sports training action classification is obtained.

This study consists of four main parts: the first part is the introduction, the second part is the fuzzy clustering model based on fuzzy vector machine optimization, the third part is the experiment and analysis, and the fourth part is the conclusion, besides there are abstracts and references.

## 2. Fuzzy Clustering Model Based on Fuzzy Vector Machine Optimization

### 2.1. Fuzzy C Division

Fuzzy C-means is used in information classification optimization. According to the diversity of information types, multilabel information is classified, and the degree of a label belonging to a certain label is described by membership degree *P*. The objective function of fuzzy C division of multilabel space *L*={*l*_1_, *l*_2_,…, *l*_*l*_} is as follows:

(1) $$\begin{array}{c} Y\left(P,c_{1},c_{2},\ldots ,c_{c}\right)=\sum_{x=1}^{c}Y_{x}=\sum_{x=1}^{c}\sum_{y=1}^{v}p_{xy}^{w}d_{xy}^{2}. \end{array}$$

Here, *p*_*xy*_ ∈ [0,1] represents the membership function of the *y*th label corresponding to the *x*th label. *P*_*xy*_ satisfies ∑_*x*=1_^*c*^*p*_*xy*_=1, and *d*_*xy*_=*l*_*y*_ − *c*_*x*_ represents all tags. *W* ∈[1,∞) represents the weighted index. The new objective function is constructed as shown in the following equation, and the necessary conditions can be obtained by calculating the minimum value of the objective function:

(2) $$\begin{array}{c} Y\left(P,c_{1},\ldots ,c_{c},\lambda_{1},\ldots ,\lambda_{y}\right)=\sum_{x=1}^{c}\sum_{y=1}^{v}p_{xy}^{w}d_{xy}^{2}+\sum_{y=1}^{v}\lambda_{y}\left(\sum_{x=1}^{c}p_{xy}-1\right). \end{array}$$

Among them, *λ*_*y*_=(*y*=1,…, *v*) represents Lagrange multipliers of *v* constraints. A derivative is taken for each input parameter, and the updated equation of membership degree is obtained as follows:

(3) $$\begin{array}{c} p_{xy}=\sum_{z=1}^{c}{\left(\frac{d_{xy}}{d_{zy}}\right)}^{\left(2/\left(w-1\right)\right)}. \end{array}$$

### 2.2. Information Gain

The information gain can quantify the relationship between random label variable *I* and *J*, and it can be calculated as follows:

(4) $$\begin{array}{c} XA\left(I,J\right)=\sum_{i\in I}^{c}\sum_{j\in J}^{v}u\left(i,j\right)\text{loa}\frac{u\left(i,j\right)}{u\left(i\right)u\left(j\right)}. \end{array}$$

Here, *u*(*i*) represents the probability density of *i*. *U* (*i*, *j*) represents the joint probability density of *i* and *j*.

Information gain can be described by combining entropy and entropy:

(5) $$\begin{array}{c} XA\left(I,J\right)=B\left(I\right)+B\left(J\right)-B\left(I,J\right). \end{array}$$

Information gain can effectively represent the degree of relationship between two or more variable labels. The larger the information gain is, the higher the relationship between variables is.

### 2.3. Dual-Label Fuzzy Vector Machine Model

Fuzzy support vector machines add a membership degree to all information samples. For multilabel information, an appropriate membership function is firstly selected according to specific problems. This function needs to be able to map the membership of all information samples *I*_*x*_ to their category *J*_*x*_. Therefore, the original training set *ζ*={(*i*_1_, *j*_1_), (*i*_2_, *j*_2_),…, (*i*_*l*_, *j*_*l*_)}. is transformed into a fuzzy training set:

(6) $$\begin{array}{c} \zeta =\left\{\left(i_{1},j_{1},\mu_{1}\right),\left(i_{2},j_{2},\mu_{2}\right),\ldots ,\left(i_{l},j_{l},\mu_{l}\right)\right\}. \end{array}$$

Among them, the *i*_*x*_ ∈ *R*^*d*^(*x*=1,2,…, *l*) represents the *x*th test information sample in *d*-dimensional space. In a multilabel classification problem, *j*_*x*_ ∈ {−1,1}, 0⩽*μ*_*x*_⩽1 represents the fuzzy membership degree of training point (*i*_*x*_*j*_*x*_*μ*_*x*_) belonging to *j*_*x*_ class.

The proposed parameter *ξ*_*x*_ is a measure of the degree of error. Fuzzy membership *μ*_*x*_ refers to the degree of training point (*i*_*x*_*j*_*x*_*μ*_*x*_) belonging to a certain class. Therefore, (*μ*_*x*_, *ξ*_*x*_) can be used to test the error fractions of training points of different importance.

For the fuzzy training set, the problem of optimal operation allocation of hyperplane is transformed into the following operation optimization problem:

(7) $$\begin{array}{c} m=C\sum_{x=1}^{l}\mu_{x}\xi_{x}-j_{x}\left(m\times i_{x}+h\right). \end{array}$$

Here, *i*_*x*_ ∈ *R*^*d*^ represents the critical degree of misclassification. *ξ*_*x*_⩾0 is the relaxation vector of the information sample. *ξ*=(*ξ*_1_, *ξ*_2_,…,*ξ*_*l*_)^*N*^. *m*, *h* represent the constant term and weight vector of linear evaluation function in the high dimensional characteristic space respectively.

The optimization problem of equation (7) is dually programmed as follows:

(8) $$\begin{array}{c} \max\sum_{x=1}^{l}\alpha_{x}=-\frac{1}{2}\sum_{y=1}^{l}\alpha_{x}\alpha_{y}j_{x}j_{y}\left(i_{x}\times i_{y}\right). \end{array}$$

The optimal solution is *α*^*∗*^=(*α*_1_^*∗*^, *α*_2_^*∗*^, ⋯,*α*_*l*_^*∗*^)^*N*^. Then, the optimal classification function is calculated as follows:

(9) $$\begin{array}{c} f\left(i\right)=\mathrm{sgn}\left\{\left(m^{*}\times i\right)+h^{*}\right\},i\in R^{d}. \end{array}$$

For nonlinear problems, adding kernel function *Z*(*i*_*x*_, *i*_*y*_) can convert formula (8) into the following equation:

(10) $$\begin{array}{c} \max\sum_{x=1}^{l}\alpha_{x}=-\frac{1}{2}\sum_{x,y=1}^{l}\alpha_{x}\alpha_{y}j_{x}j_{y}Z\left(i_{x},i_{y}\right). \end{array}$$

After calculating the optimization problem, the optimal classification function is further obtained as follows:

(11) $$\begin{array}{c} f\left(i\right)=\mathrm{sgn}\left\{\sum_{y=1}^{l}\alpha_{y}^{*}j_{y}Z\left(i,i_{x}\right)+h^{*}\right\},\;i\in R^{d}. \end{array}$$

### 2.4. Multilabel Information Classification Based on Fuzzy Vector Machine

According to the optimal classification function, the initial classification problem is decomposed into *z*(*z* − 1)/2 subproblems using a one-to-one decomposition strategy. All subproblems have multiple kinds of information. That is to say, all information samples have three situations: information samples containing only the first kind of information label, information samples containing only the second kind of information label, and samples containing both the first and second kind of information label. In order to facilitate searching, the sample containing the first information label is represented as the positive sample in the subproblem, and its output is *j*_*x*_=1. The samples containing the second information label are represented as negative samples, and the output is *j*_*x*_=−1. The samples with the first and second information labels are described as mixed samples, and the output is *j*_*x*_=0.

The optimal hyperplane is constructed by searching the support vector mechanism, and then the information samples are divided into two independent categories. However, in practical use, in some specific states, information samples cannot be completely planned into a certain category. In other words, there is a fuzzy membership relationship between samples and categories. Therefore, a fuzzy membership relationship is added through a fuzzy support vector machine to make full use of information samples.

*I*_*wt*_={(*i*_1_, *j*_1_, *μ*_1_),…, (*i*_*l*_, *j*_*l*_, *μ*_*l*_)}. Among them, *w* ∈ [1, *z*], *t* ∈ (*w*, *z*], *i*_*x*_ ∈ *R*^*d*^(*x*=1,2,…, *l*)representatives in *d* dimensions within the test information *x* samples stand for different degrees. In the multilabel classification problem, *j*_*x*_ ∈ {−1,0,1}. Among them, the total amount of information samples with the training subset information sample function value of 1, −1, and 0 is *l*^+^, *l*^−^, *l*^0^(*l*^+^+*l*^−^+*l*^0^=*l*), respectively. 0⩽*μ*_*x*_⩽1 represents the fuzzy membership degree of training point (*i*_*x*_*j*_*x*_*μ*_*x*_) belonging to some class.

The goal of the training is to find the hyperplane *f*(*i*)=*m* × *i*_*x*_+*h*, so that the positive and negative information samples can be satisfied, *m* × *i*_*x*_^+^+*h* ≥ 1, *m* × *i*_*x*_^−^+*h* ≤ −1. The information samples of the mixed class should meet the requirements of 1 > *m* × *i*_*x*_^0^+*h* > −1, so the evaluation information samples are positive classes. If +1 ≥ *f*(*i*) ≥ −1, the evaluation is mixed class. If *f*(*i*) < −1, it is evaluated as a negative class. For different types of information samples, an FSVM model was constructed based on *I*_*wt*_ on the basis of linear conditions, and the problem of hyperplane (*m* × *i*_*x*_+*h*) was calculated and transformed into a quadratic programming problem:

(12) $$\begin{array}{c} \text{wxt}\frac{1}{2}m=\varpi \left(\sum_{x=1}^{l+}\mu_{x}\xi_{x}^{+}+\sum_{x=1}^{l-}\mu_{x}\xi_{x}^{-}+\sum_{x=1}^{l}\left(\mu_{x}\xi_{x}+\mu_{x}\xi_{x}^{0-}\right)\right). \end{array}$$

Because of the different effects of training information samples on classification, the fuzzy support vector machine (FSVM) can add different misclassification penalties to different information samples and then overcome the interference of noise on classification. In the application of fuzzy support vector machine, the difficulty lies in how to determine the membership degree of the information sample. If it is determined improperly, the accuracy of the classifier will be reduced.

### 2.5. Fuzzy Vector Machine Optimization Based on Clustering

**Figure 1:**
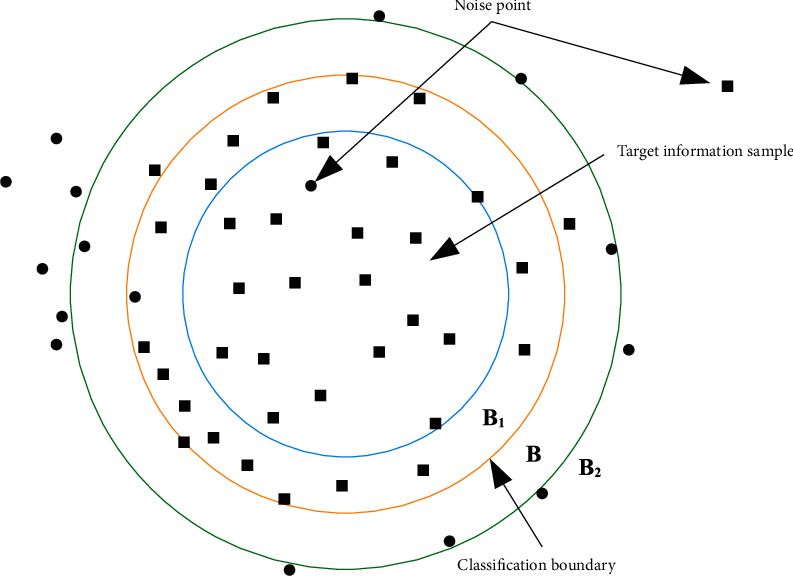
Support vector distribution.

The training efficiency of fuzzy support vector machine is slow, and most of its operations are to find the support vector and then build the optimal hyperplane. It is found that fuzzy support vector usually distributes at the edge of feature space. Most of the vectors located in the centre of the class are not support vectors and have no significance for building an optimal hyper interface. The specific process is shown in Figure 1.

In Figure 1, the black square is one type of information and the dot is another type of information. B is the optimal classified hyperplane, which is determined by a set of support vector shifts. However, there are usually some samples without reasonable explanation in the information sample, such as the noise points in the graph. These noise points will cause serious negative effects on the optimal hyperplane. In general, information samples are only part of the training set. Therefore, a more realistic approach is to delete those points that cannot be support vectors and then train the support vector machine, so as to minimize the computation and improve the optimal hyperplane results.

Because the clustering algorithm has the characteristics of high convergence efficiency, fuzzy clustering is not too sensitive to initialization problems, and its membership function can also map the close information between information samples. Therefore, the fuzzy partition method is introduced into the cluster to obtain the fuzzy clustering algorithm.

The kernel mapping Φ(*i*_*x*_) is proposed to be a nonlinear mapping of information sample *i*_*x*_ to a higher-dimensional feature space *B*. The fuzzy partition matrix is *U*={*μ*_*yn*_}, 1 ≤ *y* ≤ *C*, 1 ≤ *x* ≤ *t*.*Q*_*y*_ represents the clustering centre of the yth species cluster. *w* ∈ (1, *∞*) represents the fuzzy weighted index. Then, the dimension *d*(*i*_*x*_, *i*_*y*_) of two points *i*_*x*_ and *i*_*y*_ in the feature space is as follows:

(13) $$\begin{array}{c} d\left(i_{x},i_{y}\right)=\Phi \left(i_{x}\right)-\Phi {\left(i_{y}\right)}^{2}\\ =\Phi^{2}\left(i_{x}\right)-2\Phi \left(i_{x}\right)\cdot \Phi \left(i_{y}\right)+\Phi^{2}\left(i_{y}\right)\\ =Z\left(i_{x},i_{x}\right)-2Z\left(i_{x},i_{y}\right)+Z\left(i_{y},i_{y}\right). \end{array}$$

The concrete implementation process of fuzzy clustering method is as follows:

1. Select the termination condition of iteration *ε* ∈ (0,1), and the maximum iteration number is *N*.
2. Initialize class centre *q*_1_, *q*_2_,…, *q*_*c*_.
3. Operate *d*_*yx*_(*i*_*x*_, *q*_*y*_)=*Z*(*i*_*x*_, *i*_*x*_) − 2*Z*(*i*_*x*_, *q*_*y*_)+*Z*(*q*_*y*_, *q*_*y*_), so that *V*_*x*_=∑_*y*=1_^*c*^(1/*d*_*yx*_)^(1/*w*−1)^,  *x*=1,2,…, *t*.
4. *d*_*yu*_=min(*d*_*yx*_), if *i*_*s*_ of a certain information sample *d*_*ys*_=0, so $\mu_{yx}=\left\{\begin{array}{cc} 1, & x=s,\\ 0, & x\neq s, \end{array}\right.$. If the *d*_*yx*_ of all information samples is not zero and there exists (1/*d*_*yu*_)^(1/*w*−2)^/*V*_*u*_ ≥ *λ*, then $\mu_{yx}=\left\{\begin{array}{cc} 1, & x=p,\\ 0, & x\neq p, \end{array}\right.$. If the *d*_*yx*_ of each information sample is not equal to zero and exists simultaneously, ((1/*d*_*yu*_)^(1/*w*−2)^/*V*_*u*_ ≥ *λ*), then (*μ*_*yx*_=(1/*d*_*yu*_)^(1/*w*−2)^/∑_*y*=1_^*c*^(1/*d*_*yx*_)^(1/*w*−1)^).
5. Operate *Z*(*i*_*x*_, *q*_*y*_), *Z*(*q*_*x*_, *q*_*y*_).
6. Through the new *Z*(*i*_*x*_, *q*_*y*_), *Z*(*q*_*x*_, *q*_*y*_) return to Steps 3 and 4. Calculate the new value ${\bar{\mu }}_{yx}$ of *μ*_*yx*_.
7. If $\max\left|\mu_{yx}-{\bar{\mu }}_{yx}\right|<\varepsilon$ reached the maximum number of iterations N, then stop the operation, otherwise turn to Step 5

In the algorithm, *w* represents the parameter greater than 1. *H* is the parameter over 0, and the larger its value is, the more difficult it is to evaluate the membership degree of information sample to reach the standard of 1. After the test, when its value reaches 0.65, the desired clustering effect can be obtained. After clustering, the information samples are classified into several fuzzy classes in the form of (*i*_*x*,_*μ*_*yx*_).

Depending on the value of *μ*_*yx*_, the information sample can be divided into two categories. Information samples completely belong to a certain category, and the membership degree of such samples to other classes is 0. Such information samples are generally close to the centre of a certain class, are far from other classes and usually cannot become support vectors. The other is that the distance between information samples and other classes does not differ much, and they are located at different interface coordinates, and such samples have a certain probability to become support vectors. For the former, it can be directly classified into the closest class. There is no need to consider its association with other classes, and there is no need to enter the next support vector machine training. For the latter, it is necessary to evaluate them by training and learning and then classify them into a certain category.

## 3. Experiment and Analysis

This section begins with an introduction to the three data sets used, followed by experimental results and a discussion on different data sets. The experiments were carried out on a computer with Ubuntu 16.04 system.

### 3.1. Action Recognition Data Set

Three action recognition data sets were used in this experiment, namely, training action data set, KTH data set, and Weizmann data set. The training action data set used two GoPro Hero 7 Black with perpendicular main optical axes to shoot synchronously for 15 athletes, named as the main and secondary positions, respectively. The host camera is used to photograph the active feature plane. When performing different movements, the host position was used to photograph the sagittal or coronal plane of the athlete according to the characteristics of the movements.

**Figure 2:**
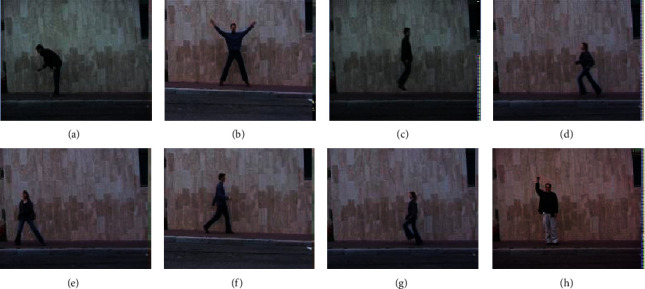
Weizmann dataset.

The KTH dataset consisted of six human movements. A total of 598 videos with an average length of 4s were shot by fixed cameras at a frame rate of 25 FPS. The Weizmann dataset contains 8 human movements. The dataset was shot by 6 subjects and contained a total of 90 videos at 50 FPS, as shown in Figure 2.

### 3.2. Experiment

#### 3.2.1. Results on Training Action Dataset

In order to validate this method on the result of recognition, on the training data set, respectively, the experiments have the following 3 parts: (1) feature vectors with different lengths are used to represent the actions on the data sets containing the shooting actions of the main and secondary engine positions respectively, and their influence on the recognition results is observed to verify the robustness of the algorithm to the changes of shooting angle and background. (2) By comparing the model in this study with literature [15], its influence on recognition results is observed. (3) Training models with different data volumes are used to observe their influence on the recognition results and verify the portability of the method on a small sample size data set.

**Figure 3:**
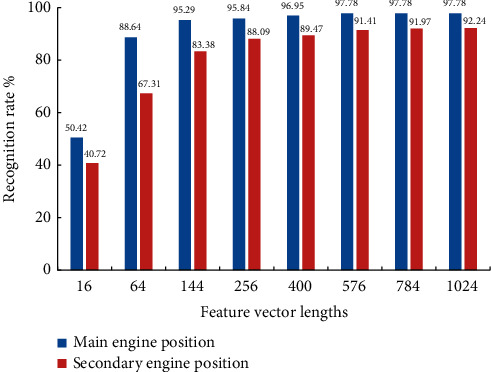
Recognition rate of different feature vector lengths.

**Figure 4:**
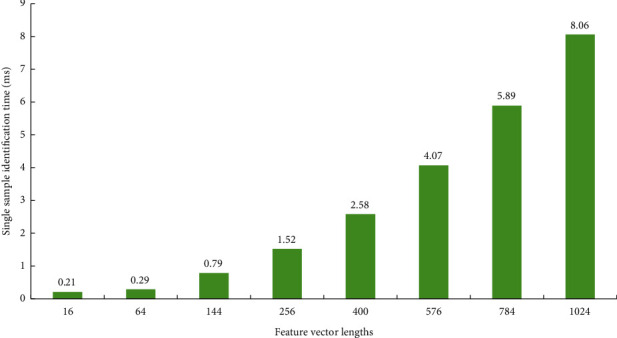
Recognition time of different feature vector lengths.

Experiments were carried out on the data set containing the action videos of primary and secondary aircraft positions. About 2,062 groups of videos of 9 people were randomly selected as the test set, and 3,792 groups of video frequency of the other 15 people were selected as the training set. Feature vectors with lengths 16, 64, 144, 256, 400, 576, 784, and 1 024 are used to represent actions, respectively. The experimental results are shown in Figures 3 and 4.

Each behavior type was filmed once or several times on nine different people, resulting in a total of 93 video sequences. Cross-validation method is adopted. In the test phase, the proposed model is evaluated frame by frame and video by video. Specifically, frame-by-frame recognition refers to the implementation of the proposed recognition algorithm for each frame and then obtaining the recognition result of each frame, and frame-by-frame recognition refers to the realization of global recognition result for the entire video sequence.

**Figure 5:**
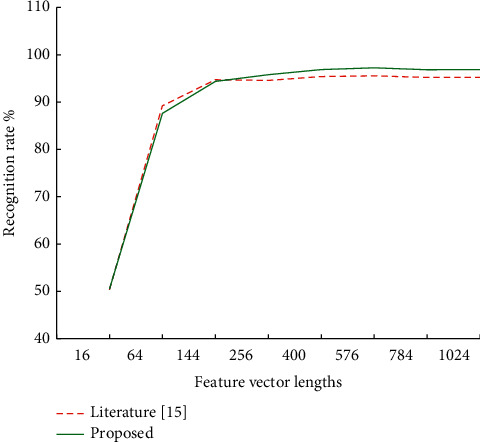
Comparison of recognition rate between proposed model and literature [15].

By comparing the recognition rates of different feature vector lengths, the feature vector with length 16 is weak in describing motion features. It is difficult to capture some motion distinguishing features located in the limbs, resulting in poor recognition results. When the length of eigenvector increases to 64, most of the actions can be recognized well. At the same time, the recognition rate of the host bit shooting action is close to 90%. When feature vectors with lengths 144 and above are used, the correct rate of identifying the main engine position is more than 95%, and the recognition rate of the secondary engine position is more than 80%. When the feature vector with the length of 576 is input as the classifier, the recognition rate of the main machine is up to 97.24%, and the recognition rate of the secondary machine is also over 90%. The recognition speed test results under feature vectors of different lengths are shown in Figure 4. The time of sample recognition changes in the same direction with the length of feature vectors, and the upward trend is obvious. The recognition speed is the same when feature vectors of the same length are used to identify the position of the main and auxiliary aircraft, so Figure 5 only shows the experimental results on the data set of the main aircraft. Based on the recognition accuracy and speed, according to the feature vector length optimization function of equation (7), the parameter *l* is determined to be 24, and the corresponding feature vector length is 576.

The recognition rate of the secondary camera position is generally lower than that of the host. The two reasons for this are as follows: (1) compared with the main feature plane of the main camera, joint occlusion is more serious in the plane of the secondary camera, which makes it more difficult to extract the motion features. (2) Different shooting backgrounds of primary and secondary aircraft positions will also affect the recognition results. In spite of this, the algorithm's recognition rate of secondary camera action can still reach 91.77%, which proves that the method has certain robustness to camera angle change and background change.

**Figure 6:**
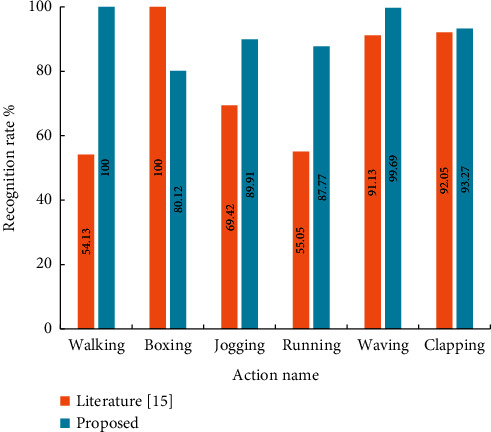
Comparison of recognition rates of proposed model and literature [15].

In order to verify the influence of the model in this study on the recognition results, the results of the model in this study and literature [15] on the recognition of training actions of host position shooting are compared. The comparison results are shown in Figure 6. Compared with literature [15], the model in this study has improved the motion recognition rate in most cases.

Because all the actions in the training action data set are executed in situ, and the overall irrelevant displacement has little impact on action recognition, the improvement of action recognition performance of this model is limited.

The influence of training set size on the recognition result. In order to verify the recognition ability of this method on the data set with a small amount of data, experiments are carried out on the training action data set photographed by the host bit. The training set containing 1, 2, 3, 7, 10, 13, and 15 action videos was used for training, and the test set containing 9 action videos was still used for testing. The experimental results are listed in Table 1. The recognition rate changes in the same direction as the amount of data in the training set. However, with the increase of data volume, the improvement of recognition accuracy by increasing training samples gradually decreases. When one person training action video (each type of action contains 1–10 samples) is used as the training set, the test can obtain 86.56% accuracy. This is because this method uses the feature vector with optimal length to represent the action. The used classifier also has a good recognition effect for small sample size data sets. The explanation has been added and marked in blue.

#### 3.2.2. Open Experimental Results on Data Sets

The test set and training set of KTH dataset are divided according to the method of literature [16]. The training set contains action videos of 16 subjects, and the test set contains action videos of 9 subjects. The method in this study is used for data processing, and the experimental results are listed in Table 2.

The comparison between the model in this study and the model recognition results in literature [15] is shown in Figure 5. The results show that for the six movements in the KTH data set, the model in this study can recognize the five movements except boxing more accurately, especially the improvement effect of walking, jogging, and running is more obvious. This is because in literature [15], the overall displacement of human body in a large range of walking, jogging, and running movements covered the effective distinguishing features between movements, and the recognition rate was greatly improved after their elimination. Finally, the recognition rate of the algorithm on THE KTH dataset reaches 91.67%, and the application of the model in this study improves the overall recognition rate by 14.

Boxing action was the only action category whose recognition rate decreased after using this method, misidentifying it as jogging or running. The reason is that the features of running and jogging movements are confused with boxing movements after eliminating a wide range of overall displacement. By observing the result of movement recognition, it is found that in the boxing movement with false detection, the punching range of the subject is usually small and the direction is horizontal, which is similar to the movement track of the upper limb of the running and jogging movements, thus causing false detection. In order to solve the confusion among individual motion categories, we can increase the weight of local motion features.

**Figure 7:**
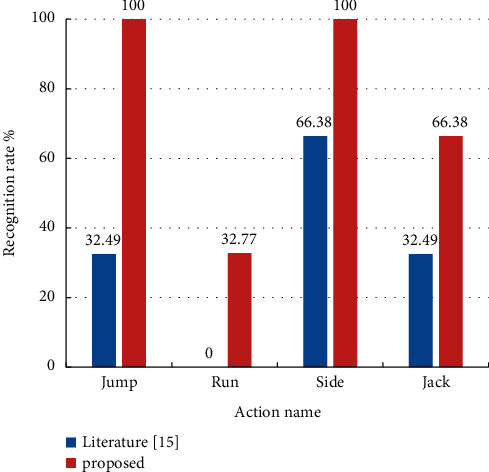
Comparison of recognition rate between proposed model and literature [15].

The Weizmann dataset was divided into a training set with 3 subjects and a test set with 3 subjects. The model in this study is compared with the model in literature [15] for data processing, training, and testing. The experimental results are listed in Table 3 and Figure 7. Except for the two actions of run and jog, the recognition rate of the other six actions is 100%, and the average recognition rate on the Weizmann data set is 90%.

The recognition rate of the four types of movements presented in Figure 7 is relatively low in literature [15]. The recognition rate of each movement is greatly improved by eliminating the overall displacement in the movement, among which the recognition rate of jump and side reaches 100%. The overall recognition rate of this dataset increased by 16.67%. Experimental results on KTH and Weizmann data sets show that the proposed algorithm has good generalization ability for the same type of data sets and can be applied to similar action recognition tasks. Although the improved projection strategy reduces the recognition rate of boxing action in the KTH data set, it greatly improves the recognition rate of all kinds of actions. In addition, the elimination of human body displacement enables the algorithm to extract relative motion features between joints to complete motion recognition, which is very important for further extraction of local motion features of human body and motion quality evaluation.

Table 4 lists the proposed algorithm and the other four algorithms on the Weizmann data set. Compared with references [17]–[20], the average accuracy per frame of the proposed method is improved by 8.05%, 4.96%, 3.74%, and 1.40%, respectively. The performance of each video recognition method is improved by 12.0%, 8.3%, 2.43%, and 0.84%, respectively. Therefore, the experimental results show that the proposed algorithm has a good result. Although the proposed method can be effective in training movements for classification, some drawbacks have been identified as the research progresses. Because all the actions in the training action data set are executed and the overall irrelevant displacement has little impact on action recognition, the improvement of action recognition performance of this model is limited.

The experimental comparison between the proposed method and other methods is listed in Table 4. In order to verify the recognition ability of the proposed method on public data sets, the proposed method is compared with other methods of the same type.  Table 5 lists the recognition results of four motion recognition methods on two public data sets, all of which are motion recognition methods based on feature extraction. The four types of methods describe the motion features by using literature [17]–[20], respectively, and complete the motion recognition by combining the classification algorithm. As listed in Table 5, the recognition accuracy of the proposed method is higher than that of other methods of the same type on the two public action recognition datasets.

## 4. Conclusion

With the rapid development of computer vision and image processing technology, the intelligent training system based on visual information processing is gradually applied to the field of sports training and rehabilitation medicine. To accurately analyze the movements of sports training using artificial intelligence techniques, an improved fuzzy clustering model is proposed in this study, which relies on the fuzzy support vector machine and clustering to complete the classification of multilabel information. The algorithm granulates the multilabel space by using fuzzy C-means, so as to obtain the correlation degree between different variable labels. Then, an appropriate membership function is selected and used to map all information samples, and the membership of its category is obtained. Then, the clustering method is used to optimize the fuzzy vector machine, establish the optimal hyperplane, and complete the classification according to their respective attributes in the high-dimensional space. Finally, the accuracy of the proposed algorithm was verified experimentally. Although the proposed method can effectively classify multilabel information, with the deepening of research, some disadvantages have been found. Therefore, the next research topic is to expand and optimize the proposed method to improve the computing power of the method.

## Figures and Tables

**Table 1:** Recognition rate comparison with different training data sizes (%).

Training data sizes	Recognition rate (%)
1	86.56
2	92.57
3	95.39
7	96.79
10	97.62
13	97.67
15	98.35

**Table 2:** Recognition rate of the proposed method on the KTH dataset.

True value	Predictive value					
	Walk	Box	Jog	Run	Wave	Applause
Walk	99	0	0	0	0	0
Box	0	80	2.5	17.5	0	0
Jog	0	0	90	10	0	0
Run	0	0	12.5	87.5	0	0
Wave	0	0	0	0	100	0
Applause	0	0	0	0	7.5	92.5

**Table 3:** Recognition rate on Weizmann dataset.

True value	Predictive value							
	Bend	Skip	Walk	Wave	Jump	Run	Side	Jog
Bend	100	0	0	0	0	0	0	0
Skip	0	100	0	0	0	0	0	0
Walk	0	0	100	0	0	0	0	0
Wave	0	0	0	100	0	0	0	0
Jump	0	0	0	0	100	0	0	0
Run	0	0	0	0	0	34.4	0	0
Side	0	0	0	0	0	0	100	0
Jog	0	0	0	0	0	0	0	66.6

**Table 4:** Comparison of recognition accuracy with different methods.

Method	Per frame (%)	Each video (%)	Frame frequency (f/s)
Literature [17]	86.99	87.98	20
Literature [18]	90.08	91.68	10
Literature [19]	91.30	97.55	30
Literature [20]	93.64	99.14	0.43
Proposed	95.04	99.98	30

**Table 5:** The comparison of recognition rate.

Datasets	Methods	Average recognition rate (%)
KTH	Literature [17]	72.94
	Literature [18]	84.56
	Literature [19]	85.42
	Literature [20]	87.58
	Proposed	92.90

Weizmann	Literature [17]	73.75
	Literature [18]	81.22
	Literature [19]	87.89
	Literature [20]	91.23
	Proposed	91.41

## Data Availability

The labeled datasets used to support the findings of this study are available from the corresponding author upon request.
